# Data-driven decision-making for precision diagnosis of digestive diseases

**DOI:** 10.1186/s12938-023-01148-1

**Published:** 2023-09-01

**Authors:** Song Jiang, Ting Wang, Kun-He Zhang

**Affiliations:** 1 Department of Gastroenterology, The First Affiliated Hospital of Nanchang University, No. 17, Yongwai Zheng Street, Nanchang, 330006 China; 2 Jiangxi Institute of Gastroenterology and Hepatology, Nanchang, 330006 China

**Keywords:** Omics data, Data-driven decision, Precise diagnosis, Machine learning, Deep learning, Digestive diseases

## Abstract

Modern omics technologies can generate massive amounts of biomedical data, providing unprecedented opportunities for individualized precision medicine. However, traditional statistical methods cannot effectively process and utilize such big data. To meet this new challenge, machine learning algorithms have been developed and applied rapidly in recent years, which are capable of reducing dimensionality, extracting features, organizing data and forming automatable data-driven clinical decision systems. Data-driven clinical decision-making have promising applications in precision medicine and has been studied in digestive diseases, including early diagnosis and screening, molecular typing, staging and stratification of digestive malignancies, as well as precise diagnosis of Crohn's disease, auxiliary diagnosis of imaging and endoscopy, differential diagnosis of cystic lesions, etiology discrimination of acute abdominal pain, stratification of upper gastrointestinal bleeding (UGIB), and real-time diagnosis of esophageal motility function, showing good application prospects. Herein, we reviewed the recent progress of data-driven clinical decision making in precision diagnosis of digestive diseases and discussed the limitations of data-driven decision making after a brief introduction of methods for data-driven decision making.

## Introduction

The concept of precision medicine has been introduced at the onset of the twenty-first century [1]. Precision medicine relies on data-driven decision-making that involves collecting massive amounts of data and organizing them to form information, and then integrating and refining the relevant information to form automated decision models via training and fitting [2]. Theoretically, with a sufficiently representative sample (data) and mathematical and statistical methods, it is possible for us to establish a model to produce prediction results that are very close to the true situation, which helps to predict the occurrence and progression of diseases and to assist in clinical diagnosis, personalized treatment and prognosis assessment [3, 4].

Human diseases involve complex and individualized pathophysiological dynamic changes, which generate big data of biology and medicine due to the increasing application of clinical examination and high-throughput biotechnologies. Therefore, current data-driven decision-making is based on the analysis of large-scale heterogeneous data [5], which is a complex process, requiring constant data input, comparing the prediction results of models with real data, then feeding deviation information to the models, and self-improving in the continuous iterative process [6].

For simple data sets, traditional statistical methods may be suitable to build models for decision-making in disease diagnosis or prognosis prediction [7, 8]. However, traditional statistical methods were not sufficient to process the large-scale heterogeneous data. Therefore, data-driven decision-making was mainly implemented through machine learning (ML) algorithms. Due to its outstanding performance, ML has been used in an increasing number of studies to process big medical data [9, 10].

With the emergence and development of multiple omics technologies, data-driven decision-making has provided a mathematical basis for the analysis of omics data in precision medicine [11], including disease diagnosis [12], prognostic assessment [13], new drug development [14], remote patient monitoring [15], bioinformatics research [16], etc.

Herein, after a brief introduction of data-driven medical decision methods, we reviewed the progress of data-driven precision diagnosis based on omics data and clinical data in digestive disease. We searched the relevant literature in PubMed database for recent 5 years. Search terms are constructed from MeSH terms, including artificial intelligence, machine learning, digestive tract diseases, digestive tract tumors, and diagnosis. A total of 629 articles were retrieved and screened individually, the closely related articles were selected for intensive reading, and the representative articles were cited in this review.

## Methods for data-driven decision-making

Data-driven decision making is achieved by ML algorithms. ML is a process in which computer learn from sample data without prior knowledge, including extracting features from the sample data, determining parameters, constructing a model and evaluating its performance, identifying and correcting deviation, and repeating the above process until the model performance cannot be improved [17]. The model can be used to predict the output values of independent external data sets [18].

Different data sets require different ML algorithms to process [7]. Traditional ML is mainly divided into unsupervised ML, supervised ML, and semi-supervised ML. Choosing an appropriate ML algorithm is critical to ensure the precision of data-driven decision-making.

Unsupervised ML is applicable for data sets without output values (labels), which can reveal hidden structures of data based on input features [19]. Main unsupervised ML methods include two types: dimensionality reduction (DR) and clustering. There are two common approaches for DR: Principal Component Analysis (PCA) [20] and t-Distributed Stochastic Neighbor Embedding (t-SNE) [21], while typical clustering algorithms include K-means clustering [22], hierarchical clustering [23], and spectral clustering [24].

Supervised ML is applicable for data sets with output values (labels), which trains a model with parameters identified during the training process to predict the output values [25]. Main supervised learning algorithms include k-nearest neighbor algorithm (KNN) [26], generalized linear model (GLM) algorithms including ordinary least squares (OLS) [27], ridge regression [28], least absolute shrinkage and selection operator (LASSO) regression [29], and logistic regression (LR) [30], Naive Bayes [31], support vector machine (SVM) [32], and random forest (RF) [33].

Semi-supervised ML trains a model based on training data set with labels to predict an unlabeled data set, and labels the unlabeled data set according to the prediction value with the highest confidence (pseudo-labeling), then incorporates the unlabeled data set with pseudo-labeling into the training data set to retrain the model until the model's prediction results remain constant [34]. Common semi-supervised ML algorithms include Self-Training, Co-Training, Transductive SVM and so on [35].

Reinforcement learning (RL) is a subfield of ML focused on how agents can learn to make sequential decisions in an environment to maximize cumulative rewards [36]. Unlike traditional ML, RL involves an agent interacting with an environment, receiving feedback in the form of rewards or penalties based on its actions. RL has been widely used in medicine [37]. Classical RL algorithms include Q-learning, Policy gradients, deep Q-networks, Actor-Critic, and Monte Carlo [38].

Deep Learning (DL) algorithms, also known as deep neural networks, are a subfield of ML that focuses on training artificial neural networks (ANN) with multiple layers, which is a further development of traditional ML algorithms [39], which has been used to process enormous data sets and surpass many classical ML methods for processing natural language, documents, images data. Deep neural networks adjust internal parameters to minimize the loss function through iteration of the backpropagation process [40]. For backpropagation, a loss function is calculated based on the difference between model output and target output, and fed back through the system, which then adjust the parameters (or weights) in each layer of the neural network to minimize the error of each neuron and the error of the entire network. Repeating above process until the error between model output and target output is minimized to acceptable levels. The principal DL algorithms include convolutional neural networks (CNN) [41], recurrent neural networks (RNN) [42], generative adversarial networks (GANs) [43], and deep reinforcement learning (DRL) [44].

## Data-driven precision diagnoses for digestive diseases

**Fig. 1:**
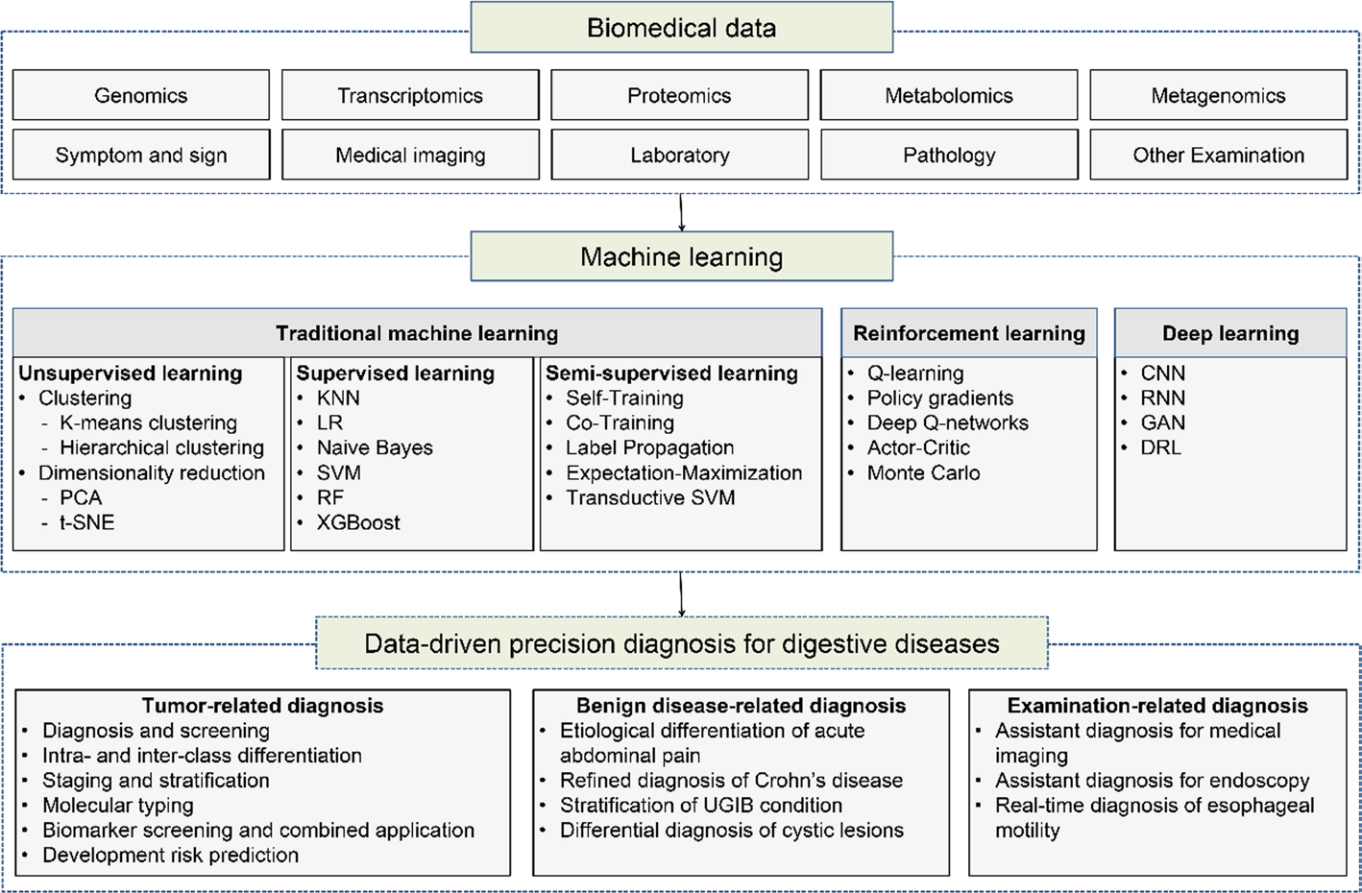
Data-driven precision diagnosis for digestive diseases. *PCA* principal component analysis, *t-SNE* t-distributed stochastic neighbor embedding, *KNN* k-nearest neighbor algorithm, *LR* logistic regression, *SVM* support vector machine, *RF* random forest, *XGBoost* extreme gradient-boosting, *CNN* convolutional neural networks, *RNN* recurrent neural networks, *GANs* generative adversarial networks, *DRL* deep reinforcement learning, *UGIB* upper gastrointestinal bleeding

Data-driven decision-making has been widely applied in medical research. Figure 1 shows the schematic diagram of data-driven decision-making in the precision diagnosis of digestive diseases.

### Data-driven precision diagnosis based on radiomics

Radiomics is a rapidly developing field of diagnostic research, which extracts quantitative metrics (features) of medical images, such as heterogeneity and shape, to inform precision diagnosis. These features can work alone or integrate with demographic, histological, genomics or proteomics data for clinical problem solving [45]. The National Cancer Institute's Quantitative Research Network has framed the radiomics in five components: (1) image acquisition and reconstruction; (2) image segmentation and mapping; (3) feature extraction and quantification; (4) database building; and (5) analysis of individual data [46].

Radiomics has shown encouraging performance in the precision diagnosis of gastrointestinal tumors. Liu et al. [47] applied radiomics to predict c-kit gene mutations in gastrointestinal stromal tumor (GIST). They collected arterial phase, venous phase, delayed phase and tri-phase combined data from contrast-enhanced CT images of 106 GIST patients, selected features with LASSO regression and GLM and then constructed a classifier using multivariate LR; the classifier showed an accuracy of 0.808 in distinguishing GIST patients with or without mutations in exon 11 of c-kit gene. This study noninvasively analyzed specific gene mutations by radiomics to support precision medicine for GIST, but it was a retrospective study and further validation is needed.

In detection of hepatic metastases of colorectal cancer (CRC), a deep learning-based lesion detection algorithm (DLLD) for CT images showed a comparable sensitivity to abdominal radiologists (81.82% vs. 80.81%) [48]. Although the DLLD had higher false-positive rate than radiologists, it may serve as an adjunct to detect liver metastases. Ma et al. extracted and selected 485 radiomic features from portal venous CT images and constructed a LASSO–Logistic regression model, which can differentiate Borrmann type IV gastric cancer (GC) from primary gastric lymphoma (PGL) [49], with an accuracy of 81.43%.

Endoscopic images have been used for data-driven precision diagnosis of gastrointestinal diseases [50]. Yasar and colleagues developed a computerized decision support system (CDS) to assist in identifying the cancerous area of endoscopic images of biopsies [51]. They assessed the performance of image segmentation algorithms in CDS, such as region growing (RG), statistical region merging (SRM), statistical region merging with region growing (SRMWRG), for detecting stomach cancerous areas, and found that RG produced the best performance, with sensitivity and specificity of 85.81% and 97.72%, respectively. CDS could help endoscopists identify cancerous areas that may have been missed and/or incompletely detected. However, data-driven precision diagnosis based on endoscopic images and videos lack of standardized imaging protocols and radiomics workflow.

Recent studies on data-driven precision diagnostics using radiomics data are summarized in Table 1.

**Table 1:** Data-driven precision diagnosis in digestive diseases based on radiomics

First author, year	Disease	*n*	Data source and specific task	ML method	Diagnostic performance	Refs.
Liu, 2022	GIST	106	Abdominal CT image; VOI segmentation, image normalization and feature extraction	GLM/LASSO	Accuracy: 80.8%	[47]
Kim, 2021	CRC	502	Abdominal CT image; ROI segmentation, feature extraction	CNN/Transfer Learning	Sensitivity: 81.82%	[48]
Ma, 2017	GC	40	Abdominal CT image; VOI segmentation, feature extraction	LASSO	Accuracy: 81.43%	[49]
Yasar, 2019	GC	10	Endoscopic image; image-based segmentation	Clustering	Accuracy: 96.33%	[51]
Li, 2021	Crohn disease	167	Abdominal CT enterography; VOI segmentation, feature extraction and selection	LASSO	AUC: 0.816 (95%CI, 0.706–0.926)	[52]
Yuan, 2022	CRC	140	Abdominal CT image; manual contouring, image-based ResNet-3D base neuron	ResNet3D/SVM	AUC: 0.922 (95%CI, 0.912–0.944)	[53]
Wu, 2022	Hepatic cystic echinococcosis	967	Abdominal ultrasound image; artificial marker repair and ROI extraction, image-based classification	DCNN	Accuracy: 90.6%	[54]
Kundu, 2020	Multi-disease detection	50	WCE image; image ROI separation, probability density function	LDA/Hierarchical SVM	Accuracy: 97.39%	[55]
Klang, 2020	Crohn disease	49	WCE image; image-based classification	CNN	Accuracy: 95.4–96.7%	[56]
Dmitriev, 2020	Pancreatic cystic lesions	134	Abdominal CT image; graph-based segmentation	RF/CNN	Accuracy: 91.7%	[57]
Meng, 2022	Crohn disease	235	Abdominal CT enterography; image ROI separation, patch-based classification	3D DCNN	AUC: 0.808–0.839	[58]
Wang, 2023	GHAC	216	Abdominal CT image; image ROI segmentation and radiomics feature extraction	LASSO	AUC: 0.731–0.942	[59]
Shi, 2023	PMME	122	Chest CT image; image resampling, tumor segmentation and feature extraction	LASSO	AUC: 0.906–0.975	[60]
Zhou, 2023	Crohn disease	316	CT enterography; VAT features extraction	PCA/LASSO/3D-CNN	AUC: 0.775 (95%CI, 0.683–0.868)	[61]
Sun, 2019	GC	100	Abdominal CT image; ROI segmentation and radiomics feature extraction	LASSO	AUC: 0.903	[62]
Lonseko, 2023	GI lesion	4880	GI endoscopic image; gastrointestinal lesion segmentation	GANs/CNN	Precision: 91.72% ± 4.05%	[63]
Jia, 2023	GIST	151	Abdominal CT image/EUS image; image segmentation, image normalization, and feature extraction	LASSO	AUC: 0.766–0.866	[64]
Guo, 2022	CRC	360	Abdominal imaging examination data; ROI segmentation and feature extraction	CNN/K-means clustering	AUC: 0.950	[65]
Du, 2023	gastric neoplasms	3449	WL and WM endoscopy image and video; ROI segmentation and feature extraction	CNN	Accuracy: 90.0%	[66]
Tang, 2023	GI tract diseases	1645	GI endoscopic image; classification and segmentation	TransMT-Net	Accuracy: 96.9%	[67]
Gong, 2023	gastric neoplasms	8993	GI endoscopic image; semantic segmentation	U-Net + + /CNN	Accuracy: 95.6%	[68]
Yang, 2023	Intestinal Metaplasia Gastritis Atrophy	21,420	Gastric endoscopic image; localization, patch-based classification	LAG/DTL	Accuracy: 97.1–99.2%	[69]
Ding, 2023	GI lesion	2565	Capsule endoscopy image and video; image-based classification	CNN/CRNN	Accuracy: 79.2–97.5%	[70]
Muniz, 2023	CRC	71	Micro-FTIR absorbance HSI from biopsy tissue; localization, voxel-based classification	FCNN/linear SVM	Accuracy: 96–99%	[71]
Du, 2023	GC	1273	Gastroscopic image; segmentation, co-spatial attention and channel attention	CSA–CA–TB–ResUnet	Accuracy: 91.2%	[72]
Yuan, 2023	GC	4315	Tongue image; patch-based classification	KNN/SVM/DT/APINet/TransFG/DeepLabV3 +	AUC: 0.830–0.920	[73]
Faust, 2023	Celiac Disease	96	Duodenitis biopsy image; CLAHE, feature extraction	SVM/KNN/DT	Accuracy: 98.5–98.6%	[74]
Kim, 2023	CRC	889	CRC histopathologic slide; patch extraction and normalization, patch-based classification	CNN	Accuracy: 95.5%	[75]
Abdelrahim, 2023	Barrett's neoplasia	270	Gastroscopy image and video; image-based classification	CNN	Accuracy: 92.0–94.7%	[76]
Fockens, 2023	Barrett's neoplasia	4920	WL endoscopy image; segmentation, image-based classification	EfficientNet‐Lite1/MobileNetV2 DeepLabV3 +	Sensitivity: 84–100%	[77]
Zhang, 2023	gastrointestinal disorders	315,767	Gastroscopy image and video; localization, video-based classification	DCNN	Accuracy: 73.1–85.2%	[78]
Zhou, 2023	gastric polyps/gastric ulcers/gastric erosions	227	Gastroscopic image; feature extraction, feature fusion, image-based classification	GoogLeNet/ResNet/ResNeXt/SVM/RF	Accuracy: 81.7–82.5%	[79]
Fan, 2023	UC	332	Endoscopic image and video; feature extraction, image-based classification	CNN	Accuracy: 86.54%	[80]
Faghani, 2022	Barrett's esophagus	542	Esophagus histology slide; image-based classification	CNN	Sensitivity: 90–100%	[81]
Yang, 2022	upper GI diseases	9403	GI endoscopic image; image-based classification	VGG-11/ResNet50/DenseNet121	Accuracy: 91.8%	[82]
Yuan, 2022	ESCC	685	GI endoscopic image; feature extraction, patch-based classification	DCNN	Accuracy: 89.8–91.3%	[83]
Luo, 2022	CAG	4005	GI WL image; image-based classification	CNN	Accuracy: 85.4–91.6%	[84]

Full names of abbreviations are given in the Abbreviations section of the manuscript

### Data-driven precision diagnostics based on genomics

With the rapid development of DNA sequencing technologies, especially whole exome sequencing (WES) and whole genome sequencing (WGS), the assessment of rare genetic mutations of complex diseases has become possible [85], facilitating the study on the pathogenesis of digestive diseases and disease diagnosis at the genetic level [86].

Genomics facilitate data-driven precise classification for GC subtype at the genetic level [87]. Based on The Cancer Genome Atlas (TCGA) database, TCGA Research Network proposed four molecular subtypes of gastric adenocarcinoma, namely, EBV-positive, microsatellite unstable, genomically stable, and chromosomally unstable tumors [88]. Ichikawa and colleagues performed a similar study, in which they identified at least one alteration in 435 cancer-related genes and 69 actionable genes of 207 patients by WES and classified GC into hypermutated and non-hypermutated tumors, and the latter was subdivided into six clusters by hierarchical clustering [89]. These molecular classifications pave the way for the molecular therapy of GC, but further studies with larger samples and multicenter clinical trials are needed.

CRC is a leading cause of cancer-related deaths globally [90], and early diagnosis plays a crucial role in improving the prognosis of patients [91]. Imperiale et al. detected multiple stool DNA targets (KRAS mutations, aberrant NDRG4 and BMP3 methylations) and used logistic-regression algorithm to build model for screening CRC [92], the combination of the stool DNA targets had a sensitivity of 92.3% for CRC and 42.6% for advanced adenomas, suggesting that multi-targeted fecal DNA screening may be an alternative test for patients who are intolerant to colonoscopy. However, the multitarget stool DNA test had more false positive results than fecal immunochemical test (FIT), and patients with positive multitarget stool DNA test require more endoscopy. Therefore, the improvement of the specificity of multitarget stool DNA test needs more attention.

Recent studies on data-driven precision diagnostics using genomics data are shown in Table 2.

**Table 2:** Data-driven precision diagnosis in digestive diseases based on genomics

First author, year	Disease	*n*	Data source and specific task	ML method	Diagnostic performance	Refs.
Ichikawa, 2017	GC	207	Tumor tissue WGS data; actionable gene-based classification	Hierarchical clustering	–	[89]
Imperiale, 2014	CRC	9989	Multitarget stool DNA testing data; multimarker-based classification	LR	Sensitivity: 92.3% Specificity: 84.6%	[92]
Luo, 2020	CRC	1822	Circulating tumor DNA methylation markers; multimarker-based classification	LASSO/RF	AUC: 0.870	[93]
Romagnoni, 2019	Crohn disease	5277	Genome-wide genotyping data; genetic variant-based classification	Penalized LR/GBT/ANN	AUC: 0.802	[94]
Chung, 2023	CMMRD	639	Low-pass genomic instability characterization (LOGIC) assay; classification based on genomic microsatellite signature	LR	Sensitivity: 100%	[95]
Zuo, 2022	PEAC	86	Tumor tissue WES and targeted bisulfite sequencing data; DNA methylation-based classification	RF/LASSO/ SVM/ XGBoost	AUC: 0.900–1.000	[96]
Wan, 2019	CRC	817	WGS data of plasma cfDNA; classification based on genetic features	PCA/SVM/ LR	AUC: 0.920 (95% CI, 0.910–0.930)	[97]
Cakmak, 2023	CRC	115	SNP profiles of immune phenotypes; prediction based on SNPs	LR/RF/SVM/KNN	AUC: 0.960	[98]
Guo, 2023	CRC	173	Tissue RNA-seq data; WGCNA, classification based on key hub genes	LASSO	AUC: 0.821–1.000	[99]
Killcoyne, 2020	EC	412	Shallow WGS data; classification based on genomic copy numbers	Elastic-net regression	Sensitivity: 72.0% Specificity: 82.0%	[100]

Full names of abbreviations are given in the Abbreviations section of the manuscript

### Data-driven precision diagnostics based on transcriptomics

The transcriptome is the sum of all RNA transcripts of an organism, including coding RNA and non-coding RNA [101]. There were two critical technologies in this field: (1) microarrays [102] for quantifying a set of specific sequences and (2) RNA sequencing (RNA-Seq) [103], which analyzes RNA transcripts with high-throughput sequencing. Transcriptomics has been widely applied for biomedical research, such as disease diagnosis and staging [104].

Patients with different stages of CRC differ in terms of therapy and prognosis. Xu et al. assessed the diagnostic capacity of tumor-educated platelet RNA profiles in differentiating CRC from healthy donors and noncancerous intestinal diseases using binary particle swarm optimization (PSO) coupled with SVM, and their classifier showed better performance than clinically utilized serum biomarkers, with areas under receiver operating characteristic curve (AUROC) ranging from 0.915 to 0.928 [105]. The tumor-educated platelet RNA profile analysis offered a potential noninvasive alternative to early CRC screening, but it was nonspecific, and related to the occurrence and development of multiple types of cancer. Zhao and coworkers identified four hub genes (BGN, COMP, COL5A2 and SPARC) based on transcriptomics and single cell sequencing, which highly expressed in GC and had potential value in diagnosis, therapy and prognosis [106]. this work, the transcriptomics data came from Gene Expression Omnibus (GEO) and TCGA databases, and thus the efficacy and generalization ability of the established diagnostic model require further verification.

There are distinct expression patterns in the transcriptomics of various tumors, including hepatocellular carcinoma (HCC) [107]. Identification of biomarkers from tumor transcriptomics could contribute to data-driven tumor diagnosis. Using different techniques to select features from large-scale transcriptomics data, Kaur et al. identified three biomarkers (FCN3, CLEC1B and PRC1) with independent diagnostic value for HCC [108] and developed diagnostic models based on the three genes with various ML algorithms (Naive Bayes, KNN, RF and LR), with diagnostic accuracies ranging from 93 to 98% and AUROCs ranging from 0.97 to 1.0 for the training and validation data sets. This study provided an alternative method for the non-invasive diagnosis of HCC; however, the research data were also derived from GEO and TCGA databases, and further validation studies are needed for the diagnostic models.

Recent reports on data-driven precision diagnostics using transcriptomics data are shown in Table 3.

**Table 3:** Data-driven precision diagnosis in digestive diseases based on transcriptomics

First author, year	Disease	*n*	Data source and specific task	ML method	Diagnostic performance	Refs.
Xu, 2022	CRC	322	Transcriptomics data of patient platelets; classification based on DEGs	SVM/PSO	AUC: 0.915–0.928	[105]
Zhao, 2021	GC	6	Transcriptomics data sets of gastric tissues; classification based on hub DEGs	Ridge regression	AUC: 0.797–0.930	[106]
Kaur, 2020	HCC	3981	Large-scale transcriptomic profiling data sets of HCC; classification based on three DEGs	Naive Bayes/RF/LR	AUC: 0.970–1.000	[108]
Sallis, 2018	EoE	193	Transcriptomics data of esophageal biopsy tissues; classification based on mRNA transcript patterns	RF/PCA	AUC: 0.985	[109]
Samadi, 2022	CRC	3523	Transcriptomic data sets from GEO database; classification based on the integration of mRNA, miRNA and lncRNA	RF/SVM/LASSO/XGBoost/CNN/BPNN	AUC: 0.885–0.999	[110]
Maurya, 2021	CRC	695	TCGA mRNA data set of CRC tissues, classification based on DEGs	LASSO/RF/KNN/ANN	Accuracy: 100%	[111]
Long, 2019	CRC	311	RNA-seq data sets of CRC from TCGA and GTEx cohorts, classification based on DEGs	RF/KNN/Naive Bayes	Accuracy: 99.8%	[112]
Sallis, 2018	EoE	215	Transcriptomics data of esophageal biopsy tissues; classification based on mRNA patterns	PCA/RF	AUC: 0.990	[113]
Su, 2022	CRC	521	TCGA transcriptomic data of CRC tissues, classification based on DEGs	RF/SVM/LASSO/DT	Accuracy: 99.81%	[114]
Lu, 2022	UC	267	Transcriptomic data sets of UC from GEO database; classification based on DEGs	LR	AUC: 0.721–0.850	[115]

Full names of abbreviations are given in the Abbreviations section of the manuscript

### Data-driven precision diagnostics based on proteomics

In the context of precision medicine, disease therapy requires individualized strategies based on latent molecular signatures to overcome the challenges arising from heterogeneity. Biological specimens, such as blood, contain abundant proteins that provide reliable information about physiological and pathological state of body [116]. Proteomics, focuses on the large-scale analysis of proteins within biological system, has promising applications in the diagnosis and personalized management of gastrointestinal diseases [117].

Esophageal cancer (EC) is one of the highly invasive cancers and the leading cause of cancer-related deaths [118]. The lack of clinically relevant molecular subtypes for EC hinders development of effective therapeutic strategies. To explore the molecular subtypes of EC, Liu et al. performed proteomics and phosphorylated proteomics profiling in 124 pairs of EC tumors and paraneoplastic tissues based on mass spectrometry (MS) [119]. Using the PCA and hierarchical clustering, they classified the EC cohort into two molecular subtypes based on protein signatures: S1 and S2. Two typical protein signatures, ELOA and SCAF4, exhibited significantly higher expression levels in the subtype S1 than in the subtype S2, and the SVM classifier developed with these two protein features yielded an AUC of 0.976 in distinguishing these two subtypes. This study provided a basis for clarifying clinically relevant molecular subtypes of EC, which could help guide subtype-based clinical treatment. However, this is a monocenter study and a multicenter trial with a large sample is still needed to validate the results.

Proteomics analysis of clinical specimens facilitates identifying protein markers and establishing non-invasive diagnostic approaches. Komor et al. performed stool proteomics to identify biomarkers for the detection of high-risk adenoma and CRC [120]. In their study, colorectal adenoma tissue samples were characterized by low-coverage WGS to determine high-risk adenomas based on specific DNA copy number changes, a LASSO regression model was built with protein biomarkers identified from proteomics data to differentiating healthy controls from patients with high-risk adenoma and CRC, the model exhibited an AUC of 0.711. Their study provided a completely noninvasive and new method for detecting high-risk adenomas that develop into CRC, but its sensitivity was low and might lead to missed diagnoses.

Recent reports on data-driven precision diagnostics using proteomics data are shown in Table 4.

**Table 4:** Data-driven precision diagnosis in digestive diseases based on proteomics

First author, year	Disease	*n*	Data source and specific task	ML method	Diagnostic performance	Refs.
Liu, 2020	EC	248	MS-based proteomic and phosphoproteomic profiles of tumor and adjacent tissues; subtyping EC based on a protein signature	PCA/clustering/SVM	AUC: 0.976	[119]
Komor, 2021	Colorectal adenomas	281	Stool proteomics data; classification based on a panel of protein biomarkers	LASSO	AUC: 0.711	[120]
Bhardwaj, 2020	CRC	259	Quantitative data of 275 plasma proteins by PEA; classification based on selected protein features	LASSO	AUC: 0.920	[121]
Kalla, 2021	IBD	552	Quantitative data of 460 serum proteins by PEA; classification based on six proteins with age and sex	LR	Accuracy: 79.8%	[122]
Demirhan, 2023	GC	64	N-glycomics data of tumor and adjacent tissues; classification by differentially expressed N-glycans	MLP	AUC: 0.980	[123]
Fan, 2022	GC	255	Urine proteomics data; classification by 4 differentially expressed urine proteins	OPLS–DA	AUC: 0.810–0.920	[124]
Bergemalm, 2021	UC	451	Quantitative data of 92 plasma proteins by PEA; preclinical prediction by a panel of up-regulated proteins	PCA/LR	AUC: 0.920	[125]
Zhao, 2020	Acute appendicitis	568	Urinary proteomics data; classification based on a 10-protein signature	RF/SVM/Naive Bayes	Accuracy: 81.2–83.6%	[126]
Song, 2020	GC	60	Label-free global proteomics data of tumor and control tissues; classification based on a four-protein signature	RF	AUC: 0.886–0.996	[127]
Shen, 2019	GC	150	Targeted proteomics data of serum by PEA; classification based on 19 proteins	Elastic-net regression	AUC: 0.990	[128]
Chatziioannou, 2018	NEC	86	Serum proteomics profiles; classification based on two panels of three proteins	OPLS–DA	AUC: 0.999	[129]

Full names of abbreviations are given in the Abbreviations section of the manuscript

### Data-driven precision diagnostics based on metabolomics

Metabolomics refers to comprehensive and simultaneous analysis of metabolites in biological samples and estimate their effective changes triggered by various conditions for instance, diet, lifestyle, genetic or environmental factors [130]. Due to inherent sensitivity of metabolomics, subtle changes of biological pathways can be detected, providing insight into the mechanisms hidden under various physiological conditions and abnormal processes [131].

Gastrointestinal system is the most central metabolic organ [132], and changes of intestinal bacterial content (intestinal microecological dysbiosis) and disruption of intestinal epithelial barrier can induce or exacerbate disease [133]. Jiménez and colleagues analyzed metabolite spectra of cancerous and para-carcinoma tissues from CRC patients using high-resolution magic angle spinning nuclear magnetic resonance (HR–MAS–NMR) and showed significant biochemical differences between two types of tissues [134], the metabolic profile of tumor tissues can distinguish tumors at different T and N stages, suggesting that it may have value in tumor staging. However, the sample size of the study was small, and further validation studies are warranted.

Lipid omics is a branch of metabolomics that targets lipid metabolites and has been used to identify biomarkers for tumor. Yuan et al. performed a lipidomic analysis in 525 serum samples and developed a diagnostic model containing 12 lipid biomarkers and age and gender by ML [135], which performed well for detecting esophageal squamous cell carcinoma (ESCC) with AUC of 0.958, 0.966 and 0.818 and sensitivities of 90.7%, 91.3% and 90.7% in the training, validation and independent validation cohorts, respectively. However, despite its good diagnostic efficiency, the model contains many variables and needs further optimization to improve its utility.

Metabolomics may play an important role in the differential diagnosis based on clinical symptoms. Takis et al. performed proton nuclear magnetic resonance (^1^H-NMR) spectroscopy of serum to extract individual metabolic fingerprints in two groups of patients who suffered from different acute abdominal pain (epigastric pain vs. diffuse abdominal pain) [136] and showed that metabolomics fingerprint could distinguish two groups of patients with high accuracy (> 90%); further analysis demonstrated that metabolomics fingerprint could distinguish the etiology of abdominal pain in the two groups with accuracies of > 70% and > 85%. These findings indicate that serum metabolomics may help emergency physicians to diagnose acute abdominal pain precisely. Non-targeted MRI-based metabolomics for the diagnosis of acute GI diseases has the advantages of being rapid, accurate and non-invasive, but its practical value needs to be further investigated.

Recent reports on data-driven precision diagnostics using metabolomics data are shown in Table 5.

**Table 5:** Data-driven precision diagnosis in digestive diseases based on metabolomics

First author, year	Disease	*n*	Data source and specific task	ML method	Diagnostic performance	Refs.
Jiménez, 2013	CRC	26	Metabolic profiles of tumor and adjacent tissues by NMR spectroscopy; classification based on discriminant metabolites	OPLS–DA	AUC: 0.910	[134]
Yuan, 2021	ESCC	525	Serum lipidomics data; classification based on a panel of 12 lipid biomarkers, age and gender	SVM/PCA	AUC: 0.818–0.966	[135]
Takis, 2018	Diffuse abdominal pain	64	Serum metabolomics data by NMR spectroscopy; classification by metabolomics fingerprint	OPLS–DA/PCA	Accuracy > 90%	[136]
Wang, 2023	ESCC	1104	Serum metabolomics data by LC–MS; classification based on digital images of metabolome profiles	CNN	AUC: 0.950	[137]
Yang, 2022	CRC	99	LC–MS-based plasma lipidomics data; classification based on 14 lipids	PLS/RF/SVM/KNN	Accuracy: 72.6–100%	[138]
Huang, 2021	GC	400	Untargeted metabolomics data of plasma; classification based on 6 metabolites with clinical indicators	LR/RF	AUC: 0.830	[139]
Yu, 2023	GC	301	Serum metabolomics data by MS; classification based on 12 differential metabolites	PCA/SVM/RF/LASSO	AUC: 0.893	[140]
Matsumoto, 2023	GC	101	Hydrophilic metabolites quantified by LC–TOFMS; classification based on 3 metabolites	SVM	AUC: 0.885–0.915	[141]
Pan, 2022	GC	280	Target bile acid metabolomics data of serum; classification based on 6 bile acids	RF/LASSO/OPLS–DA	AUC: 0.940–1.000	[142]
Zhao, 2022	ESCC	239	Multi-platform metabolomics data of serum; classification based on 5 metabolites	RF/LASSO/PCA	AUC: 0.873 (95% CI, 0.825–0.925)	[143]

Full names of abbreviations are given in the Abbreviations section of the manuscript

### Data-driven precision diagnostics based on metagenomics

Intestinal microbiome is a microbial ecosystem that expresses 100 times greater number of genes than human hosts and plays a critical role in human health and disease pathogenesis [144]. Next generation sequencing technologies, such as 16S rRNA, internal transcribed spacer (ITS) sequencing, metagenomics sequencing and viral sequencing [145], have been widely applied to the study of intestinal microbiome. Traditional techniques for metagenomics depend on prior knowledge [146, 147] and are unable to annotate sequences not available in database [148]. In recent years, innovative approaches based on traditional ML and DL algorithms have emerged to analyze metagenomics data [149]. For example, unsupervised or supervised learning models were widely applied for classification or clustering of samples based on annotation matrices [150, 151].

Metagenomics-based precision medicine has become a hot topic in gastrointestinal disease research. Nonalcoholic fatty liver disease (NAFLD) is an important etiology of chronic liver disease, which can lead to liver cirrhosis (LC), HCC and liver-related death [152]. Loomba et al. used gut microbial metagenomics to distinguish liver fibrosis levels in NAFLD patients [153]. They characterized the composition of gut microbiome by metagenomics sequencing of DNA extracted from stool samples and constructed a RF classifier containing 40 features that distinguished liver fibrosis between stages 0–2 and stages 3–4 with an AUC of 0.936. This study, which detects the level of liver fibrosis in NAND from the perspective of intestinal microbiome, is an interesting study that deserves further validation. Yang et al. performed a metagenomics analysis of the intestinal microbiome of 52 CRC patients and 55 healthy family members and found significant differences between the gut microbiomes of CRC patients and healthy family members and constructed an RF classifier with 22 microbial genes that could accurately distinguish CRC patients from healthy controls with an AUC of 0.905, 0.811, 0.859 in Chongqing, Hong Kong and French cohorts, respectively [154], which may be valuable for the early CRC diagnosis. However, it is not known whether this method can distinguish CRC from benign intestinal diseases.

Recent reports on data-driven precision diagnostics using metagenomics data are shown in Table 6.

**Table 6:** Data-driven precision diagnosis in digestive diseases based on metagenomics

First author, year	Disease	*n*	Data source and specific task	ML method	Diagnostic performance	Refs.
Loomba, 2017	NAFLD	86	Gut metagenomics data of stool; classification based on a fecal metagenomic signature	RF/SVM/clustering	AUC: 0.936	[153]
Yang, 2020	CRC	534	Fecal metagenomics data; classification based on fecal microbiomics biomarkers	Clustering/RF	AUC: 0.811–0.930	[154]
Bang, 2019	CRC	404	Gut microbiome data from 16 S rRNA sequencing; classification based on gut microbiome	SVM/KNN/LogitBoost	Accuracy: 96.84%	[155]
Dai, 2018	CRC	526	Gut metagenomics data; classification based on seven CRC-enriched bacterial markers	PCA/SVM	AUC: 0.820–0.84	[156]
Abbas, 2019	IBD	973	Gut metagenomics data of biopsy samples from QIITA database; classification based selected features by NBBD	RF	AUC: 0.760–0.800	[157]
Syama, 2023	CRC/IBD	1849	Gut metagenomics data sets of CRC and IBD; classification based on gut metagenomics data by boosting GraphSAGE	GCN	AUC: 0.900–0.930	[158]
Lee, 2022	IBD/CRC/LC	644	Gut metagenomics data sets; classification based on metagenome features	RF/SVM/PCR/LASSO/XGBoost/	AUC: 0.840–0.980	[159]
Forbes, 2018	UC	102	Gut metagenomics data; classification based on abundant taxonomic biomarkers of gut microbiota	Naive Bayes/RF/PCA	AUC: 0.900–0.930	[160]
Liang, 2020	CRC	1012	Fecal metagenomics data; classification based on combining several gut microbial gene markers with FIT	LR	Sensitivity: 93.8% Specificity: 81.2%	[161]
Hollister, 2019	IBS	45	Fecal metagenomics and metabolomics data; classification based on fecal metagenomic and metabolic markers	RF/LASSO/SVM/Naive Bayes	AUC: 0.930	[162]

Full names of abbreviations are given in the Abbreviations section of the manuscript

### Data-driven precision diagnosis based on clinical data

Daily clinical practice generates medical big data involving disease history, laboratory examinations, medical images, pathology, therapy, etc. ML algorithms can mine more information from medical big data to facilitate precision diagnosis. For example, the development of diagnostic models based on clinical big data sets can provide clinicians with data-driven decision-making advice, thereby facilitating the evolution from guideline-oriented medicine to individualized precision medicine.

Laboratory data are frequently used in data-driven diagnostics based on clinical data. Li et al. developed diagnostic models based on the data of traditional laboratory examinations to detect CRC [163]. They extracted laboratory data, including liver enzymes, lipids, complete blood counts and tumor biomarkers from electronic medical records of patients with CRC and healthy controls, and applied five ML algorithms (LR, RF, KNN, SVM and Naive Bayes) to develop diagnostic models for CRC, in which the LR model performed best for identifying CRC, with AUC 0.865, sensitivity 89.5%, specificity 83.5%, PPV 84.4%, and NPV 88.9%.

Combining multiple types of clinical data may be necessary for data-driven diagnosis in certain conditions. Hu et al. performed a precision diagnostic study in patients initially diagnosed as gastric GIST [164]. They collected multiple types of preoperative data of the patients, including hematological indicators, features of enhanced CT and ultrasonic gastroscopy, and then developed and validated a diagnostic model for differentiating GIST from other confusing tumors by extreme gradient-boosting (XGBoost) algorithm, with an accuracy of 73%.

The use of routine clinical examination data to build valuable diagnostic models should be valued, as the data are derived from routine clinical work without additional testing.

Recent reports on data-driven precision diagnostics using clinical data are shown in Table 7.

**Table 7:** Data-driven precision diagnosis in digestive diseases based on clinical data

First author, year	Disease	*n*	Data source and specific task	ML method	Diagnostic performance	Refs.
Li, 2021	CRC	1164	Laboratory test data from electronic medical records; classification based on four laboratory indicators	LR/RF/KNN/SVM/Naïve Bayes	AUC: 0.849 (95%CI, 0.840–0.860)	[163]
Hu, 2021	GIST	124	Clinical examination data of pre-operation; classification based on CT and EUS features	XGBoost	AUC: 0.770 (95%CI, 0.570–0.900)	[164]
Shung, 2020	UGIB	2357	Clinical and laboratory indicators; classification based on variables of demography, comorbidity, clinical feature and laboratory	XGBoost	AUC: 0.90 (95%CI, 0.87 − 0.93)	[165]
Wang, 2021	Esophageal motility function	229	Esophageal HRM data sets; predicting esophageal motility function over HRM features	Conv3D/BiConvLSTM	Accuracy: 91.32%	[166]
Zhu, 2020	GC	709	Demographic and laboratory indicators from electronic medical records; classification based on a panel of independent predictors	GBDT	Accuracy: 83.0%	[167]
Phan-Mai, 2023	Complicated Appendicitis	1950	Medical record data; classification based on indicators of demography, blood test, and ultrasound of the appendix	SVM/DT/LR/KNN/ANN/GB	AUC: 0.64–0.89	[168]
Nemlander, 2023	CRC	2681	PHC data; classification based on diseases diagnosed in PHC consultations and consultation number	SGB/LR	AUC: 0.830 (95%CI, 0.790–0.870)	[169]
Popa, 2022	EMD	157	Esophageal HRM images; classification based on the images	CNN	Accuracy: 93.0%	[170]
Fan, 2022	GC	574	Medical record data; classification based on age, sex and classical serum tumor markers	LR/RF	Accuracy: 86.8%	[171]
Kou, 2022	EMD	1741	Esophageal HRM data set; classification based on raw multi-swallow data	CNN/ANN/XGBoost/Bayes	Accuracy: 88.0–93.0%	[172]
Ho, 2023	EC	819	Questionnaire data from the SPIT and RISQ data sets; classification based on 17 features selected from questionnaire response	LDA/GLMNET/SVM/RF/KNN/CART/GLM	AUC: 0.710–0.920	[173]

Full names of abbreviations are given in the Abbreviations section of the manuscript

### Data-driven precision diagnostics based on integrated omics

Due to nonlinear interactions and joint effect of multiple factors generated from biological systems, it became difficult to discern true biological signal from random noise. Noise may come from biological systems, analytical platforms, and various data-specific analytical workflows, which complicates the integration of data across omics. Nevertheless, integrated omics and clinical data provide more comprehensive and valid information that facilitate precision medicine [174].

A combined multi-omics analysis can provide a better molecular classification of tumors. Liu et al. used clustering approach to analyze the data sets of gene copy number alterations (CNAs), DNA methylation, mRNA and miRNA and divided 256 HCC samples into five subgroups, each showing distinct survival rates and molecular signature [175].

Integrated analysis of multiple omics can provide better diagnostic performance. Al-Harazi et al. established and validated a new network-based approach to analyze CRC [176], in which they performed an integrated analysis of whole genome gene expression profile and CNAs data sets to construct a gene interaction network for each significantly altered gene, and then these gene interaction networks were clustered to form gene interaction subnetwork markers. Using these subnetwork markers, a SVM classifier based on 15 subnetwork markers were developed, which showed over 98% accuracy in detecting CRC patients, providing better value for disease diagnosis compared to individual gene markers.

Diagnostic methods based on multi-omics can reveal the heterogeneity of gastrointestinal tumors, which facilitates physician to more fully understand the genetic differences of individual patients and develop targeted therapies. However, they are cumbersome in steps, difficult to collect data and generalize.

Recent reports on data-driven precision diagnostics using multi-omics data are shown in Table 8.

**Table 8:** Data-driven precision diagnosis in digestive diseases based on integrated omics

First author, year	Disease	*n*	Data source and specific task	ML method	Diagnostic performance	Refs.
Liu, 2016	HCC	256	CNAs, DNA methylation, mRNA, and miRNA data from TCGA; subtyping HCC by multi-omics data	PCA/LR/Clustering	AUC: 0.780–1.000	[175]
Al-Harazi, 2021	CRC	89	Whole-genome gene expression profiling and CNA data sets from GEO database; classification based on the cores of 15 subnetwork markers	SVM/PCA/Clustering	Accuracy: 98.0%	[176]
Hoshino, 2022	CRC	24	Radiomics data of CT image and DNA sequencing data of tumor mutation burden; prediction of tumor mutation burden based on the image features	RF/XGBoost	Accuracy: 68.2%	[177]
Gawel, 2019	CRC	160	Public proteomics and transcriptomics data sets of tumor and adjacent tissues; classification based on nine secreted protein markers	Random elastic net	Sensitivity: 90.0% Specificity: 92.0%	[178]
Gai, 2023	CAG/GC	319	Fecal metabonomics and microbiota profiles data; classification based on 2 fecal metabolites and 2 gut microbes	SVM/RF	AUC: 0.88 Accuracy: 85.7%	[179]
Huang, 2022	CRC	743	Genomic and epigenetic profiles data sets of tissues from TCGA and GEO databases; classification based on DNA methylation and mutation burden data	LASSO/SVM/PCA/LR	AUC: 0.857–1.000	[180]
Cao, 2020	CRC	1214	Pathomics, genomic and transcriptomic data sets; classification base on pathomics signature	Residual CNN/XGBoost/Naive Bayes	AUC: 0.850–0.885	[181]
Gonzalez, 2022	Crohn disease	182	Fecal metaproteomics, metagenomics, metabolomics, and host genetics data; prediction of CD location based on a multi-omics feature set from metabolomics and metaproteomics	RF/LR/ExtraTrees/DT/Naive Bayes/KNN/SVC/MLPC/Voting Classifier/Adaboost	AUC: 0.94	[182]
Adel-Patient, 2023	EoE	32	Tissue transcriptomics, tissue and blood immunologic components, and plasma metabolomics data sets; classification based on combining plasma metabolomics and cytokine biomarkers	PLS–DA/PCA	AUC: 0.929	[183]
Xing, 2023	CRC	212	Tissue transcriptomics and plasma metabolomics data; classification based on combining metabolomics and RNA-seq data	PLS–DA/PCA	AUC: 0.904–0.923	[184]
Kel, 2019	CRC	202	Full genome gene-expression data and genomic CpG island methylation data from tumor and gut epithelial tissues; classification based on 6 hypermethylated gene markers	F-Match/CMAcorrel/SVM/master-regulator search algorithm	Accuracy: 92.3%	[185]
Ding, 2019	CRC	315	Transcriptomics and proteomics data of CRC; classification based on secreted biomarkers	SVM	Accuracy: 85.9%	[186]

Full names of abbreviations are given in the Abbreviations section of the manuscript

## Limitations and prospects of data-driven decision making

With the development of biological technology and computer science, the cost of acquiring omics data and time required to analyze and process them have been significantly reduced. The application of ML algorithms to study the intrinsic patterns and correlations of medical data for data-driven disease diagnosis and prediction has become a research hotspot. Many clinical trials of data-driven clinical decision-making systems based on ML and medical big data have been registered. For instance, Wallace MB et al. compared adenoma miss rate of colonoscopy with GI-Genius (Medtronic), which has been currently approved as a medical device in both the United States and the European Union, and found that AI reduced adenoma miss rate by about twofold [187]. Another randomized controlled trial to develop and validate the Gastrointestinal Artificial Intelligence Diagnostic System (GRAIDS) for the diagnosis of upper gastrointestinal cancers has been conducted in six hospitals of different tiers in China [188], and the results showed that GRAIDS had high diagnostic accuracy in detecting upper gastrointestinal cancer, with sensitivity similar to that of endoscopists, better than that of non-expert endoscopists.

However, there are still some issues that need to be addressed in the medical application of data-driven decision making. Although many reports on various models of data-driven decision making have been reported, few of them are applied in clinical practice. One of the reasons may be that the low quality of the data sets used to build ML models affects their practical application. Low-quality data sets can seriously impact the accuracy of data-driven decisions, the so-called garbage in, garbage out. Thereby, a prerequisite for effective data-driven decision making is to build high quality, well-constructed data sets. High-quality data sets can improve the predictive ability of ML algorithms and meanwhile reduce the size of data sets required for training models and the complexity of data representation. In addition, the ML models built for data-driven decision making need to be rigorously evaluated and optimized, which also requires new high-quality data sets to validate their application value and generalization performance.

The ML-based data-driven methodology still has some limitations. First, a critical drawback of DL algorithms is that it requires large amounts of data to train deep neural networks, and such scaled data sets are usually unachievable for many medical studies. Second, the interpretation of complex ML algorithms remains problematic. Third, considering the demand for large-scale data sets for data-driven, it is usually a challenge to integrate data sets across different platforms, languages and countries. Besides, the annotation of data sets from different sources differs, thus a uniform, standardized and publicly accepted data annotation system is required. An important point to remember is that classical ML algorithms require much less data than DL-based strategies; therefore, analyzing non-big data by appropriate classical ML algorithms can also be useful in precision medicine.

Although a data-driven diagnostic system can facilitate clinical decision-making, it can only provide physicians with complementary advice to assist them in noticing problems they tend to overlook, not replace them in making diagnostic decisions. Excessive dependency of advice from a data-driven decision-making system is detrimental to the training of young physicians. Due to advances in science and technology, traditional physical examinations have been reduced and replaced by examinations performed by machines in modern medical practice, which led patients to doubt the competence of their physicians, and this distrust will increase if the patients are informed that the diagnosis comes from the computer.

Therefore, many aspects need to be improved before data-driven diagnostic systems become available for routine clinical application, including the establishment of high-quality data sets, standardization of data sets from different sources, selection of appropriate ML algorithms, improvement of relevant laws and regulations, and education for physicians and patients.

## Conclusion

Mining the clinical value of medical data to build a data-driven medical decision-making system is a current research hotspot, which is important for large-scale medical data that are difficult for the human brain to process. In the data processing, there is no clear boundary between ML and traditional statistical approaches [189]. In general, traditional statistical models may perform better than ML algorithms for simple data sets, while for complex data sets and specific objectives, ML algorithms are required. Studies on data-driven medical decision making in digestive diseases have mainly focused on tumors, including detection and screening, molecular typing, staging, stratification, intra- and inter-class discrimination, as well as risk prediction. There are also reports on data-driven diagnosis and therapy for gastrointestinal non-tumor diseases, such as etiology differentiation of acute abdominal pain, precise diagnosis of Crohn's disease, stratification of UGIB, and real-time diagnosis of esophageal motility. Although data-driven clinical decision-making can contribute the precision of diagnosis of digestive diseases, there are still some limitations that need to be improved, including the establishment of high-quality data sets, standardization of data sets from different sources, selection of suitable ML algorithms, completion of relevant laws and regulations, relevant education for physicians and patients. However, it is believed that as relevant research continues to progress, data-driven clinical decision-making systems will be increasingly used in clinical practice and will become important assistants to clinicians and contribute to precision medicine.

## Data Availability

Not applicable.

## Abbreviations

ANN: Artificial neural networks
APINet: Attentive pairwise interaction neural network
AUROC: Areas under receiver operating characteristic curve
BiConvLSTM: Bidirectional convolutional long–short-term-memory
BPNN: Back propagation neural network
CAG: Chronic atrophic gastritis
CART: Classification and regression tree
CDS: Computerized decision support system
CLAHE: Contrast Limited Adaptive Histogram Equalization
CMMRD: Constitutional mismatch repair deficiency
CNAs: Copy number alterations
CNN: Convolutional neural networks
Conv3D: Three-dimensional convolution
CRC: Colorectal cancer
CRNN: Convolutional recurrent neural network
CSA-CA–TB–ResUnet: Co-spatial attention and channel attention-based triple-branch ResUnet
3D DCNN: Three-dimensional deep convolutional neural networks
3D-CNN: Three-dimensional convolutional neural networks
DCNN: Deep convolutional neural networks
DEG: Differentially expressed gene
DL: Deep learning
DLLD: Deep learning-based lesion detection algorithm
DR: Dimensionality reduction
DRL: Deep reinforcement learning
DT: Decision trees
DTL: Dual transfer learning
EA: Ensemble algorithms
EC: Esophageal cancer
EMD: Esophageal motility disorders
EoE: Eosinophilic oesophagitis
ESCC: Esophageal squamous cell carcinoma
EUS: Endoscopic ultrasonography
FCNN: Fully connected neural network
FIT: Fecal immunochemical test
FTIR: Fourier transform infrared
GANs: Generative adversarial networks
GB: Gradient boosting
GBDT: Gradient boosting decision tree
GBT: Gradient boosted trees
GC: Gastric cancer
GCN: Graph convolutional network
GEO: Gene Expression Omnibus
GHAC: Gastric hepatoid adenocarcinoma
GI: Gastrointestinal
GIST: Gastrointestinal stromal tumor
GLM: Generalized linear model
GLMNET: Generalized linear model NETworks
GO: Gene Ontology
GRAIDS: Gastrointestinal Artificial Intelligence Diagnostic System
GraphSAGE: Graph SAmple and aggregate
GTEx: Genotype-Tissue Expression
HCC: Hepatocellular carcinoma
HSI: Hyperspectral imaging
^1^H-NMR: Proton nuclear magnetic resonance
HRM: High-resolution manometry
HR-MAS-NMR: High-resolution magic angle spinning nuclear magnetic resonance
IBD: Inflammatory bowel disease
IBS: Irritable bowel syndrome
ITS: Internal transcribed spacer
KNN: K-nearest neighbor algorithm
LAG: Local attention grouping
LASSO: Least absolute shrinkage and selection operator
LC: Liver cirrhosis
LC–MS: Liquid chromatography with mass spectrometry
LC–TOFMS: Liquid chromatography–time-of-flight mass spectrometry
LDA: Linear discriminant analysis
LOGIC: Low-pass genomic instability characterization
LR: Logistic regression
ML: Machine learning
MLP: Multilayer perceptron algorithm
MLPC: Multilayer perceptron classifier
MS: Mass spectrometry
NAFLD: Nonalcoholic fatty liver disease
NBBD: Network-Based Biomarker Discovery
NMR: Nuclear magnetic resonance
OLS: Ordinary least squares
OPLS–DA: Orthogonal projection to latent structures–discriminant analysis
PCA: Principal component analysis
PCR: Principal component regression
PEA: Proximity extension assay
PEAC: Pulmonary enteric adenocarcinoma
PGL: Primary gastric lymphoma
PHC: Primary health care
PLS: Partial least squares
PLS–DA: Partial least square–discriminant analysis
PMME: Primary malignant melanoma of the esophagus
PSO: Particle swarm optimization
RF: Random forest
RG: Region growing
RISQ: Predicting risk of disease using detailed questionnaires
RL: Reinforcement learning
RNA-Seq: RNA sequencing
RNN: Recurrent neural networks
ROI: Region of interest
SGB: Stochastic gradient boosting
SNPs: Single nucleotide polymorphisms
SPIT: The saliva to predict risk of disease using transcriptomics and epigenetics
SRM: Statistical region merging
SRMWRG: Statistical region merging with region growing
SVC: Support Vector Classification
SVM: Support vector machine
TCGA: The Cancer Genome Atlas database
TransFG: Transformer architecture for fine-grained recognition
t-SNE: T-distributed stochastic neighbor Embedding
UC: Ulcerative colitis
UGIB: Upper gastrointestinal bleeding
VAT: Visceral adipose tissue
VOI: Volume of interest
WCE: Wireless capsule endoscopy
WES: Whole exome sequencing
WGCNA: Weighted gene co-expression network analysis
WGS: Whole genome sequencing
WL: White light
WM: Weak-magnifying
XGBoost: Extreme gradient-boosting
